# Development of a Novel Metagenomic Biomarker for Prediction of Upper Gastrointestinal Tract Involvement in Patients With Crohn’s Disease

**DOI:** 10.3389/fmicb.2020.01162

**Published:** 2020-06-03

**Authors:** Min Seob Kwak, Jae Myung Cha, Hyun Phil Shin, Jung Won Jeon, Jin Young Yoon

**Affiliations:** Department of Internal Medicine, Kyung Hee University Hospital at Gangdong, College of Medicine, Kyung Hee University, Seoul, South Korea

**Keywords:** microbiome, 16S rRNA, Crohn’s disease, upper gastrointestinal tract, biomarker

## Abstract

The human gut microbiota is an important component in the pathogenesis of Crohn’s disease (CD), promoting host–microbe imbalances and disturbing intestinal and immune homeostasis. We aimed to assess the potential clinical usefulness of the colonic tissue microbiome for obtaining biomarkers for upper gastrointestinal (UGI) tract involvement in CD. We analyzed colonic tissue samples from 26 CD patients (13 with and 13 without UGI involvement at diagnosis) from the Inflammatory Bowel Disease Multi-Omics Database. QIIME1, DiTaxa, linear discriminant analysis effect size (LEfSe), and PICRUSt2 methods were used to examine microbial dysbiosis. Linear support vector machine (SVM) and random forest classifier (RF) algorithms were used to identify the UGI tract involvement-associated biomarkers. There were no statistically significant differences in community richness, phylogenetic diversity, and phylogenetic distance between the two groups of CD patients. DiTaxa analysis predicted significant association of the species *Ruminococcus torques* with UGI involvement, which was confirmed by the LEfSe analysis (*P* = 0.025). For the feature ranking method in both linear SVM and RF models, the species *R. torques* and age at diagnosis contributed to the combined models. The L-methionine biosynthesis III (*P* = 0.038) and palmitate biosynthesis II (*P* = 0.050) were under-represented in CD with UGI involvement. These findings suggest that *R. torques* might serve as a novel potential biomarker for UGI involvement in CD and its correlations, in addition to a range of bacterial species. The mechanisms of interaction between hosts and *R. torques* should be further investigated.

## Introduction

Crohn’s disease (CD) is a heterogeneous disorder with a multifactorial etiology, including genetic factors, host immune system, environmental factors, and gut microbiota, and is characterized by chronic relapsing transmural inflammation which can affect the gastrointestinal tract (Strober et al., 2007). It may affect any part of the gastrointestinal tract, from the mouth to the perianal area, although the terminal ileum and the right colon are the most commonly affected sites (Strober et al., 2007; Gajendran et al., 2018). Previous studies estimated the prevalence of CD patients affected with an upper gastrointestinal (UGI) tract involvement at 16–34% for adults (Lenaerts et al., 1989; Cameron, 1991; Kefalas, 2003; Castellaneta et al., 2004; Van Limbergen et al., 2008) and 26–54% for children (Annunziata et al., 2012; Diaz et al., 2015). The UGI tract involvement in CD represents a risk of complications, such as stricturing and fistulizing phenotypes (Bernell et al., 2000), recurrence (Wolters et al., 2006), further hospitalization (Chow et al., 2009), and surgery (Bernell et al., 2000; Wolters et al., 2006; Henriksen et al., 2007; Lazarev et al., 2013; Davis, 2015; Gomollon et al., 2017).

Accordingly, the European Crohn’s and Colitis Organization consensus guideline recommends that UGI endoscopy and radiology, such as magnetic resonance imaging, computed tomography, and small bowel capsule endoscopy, should be performed in all CD patients where UGI tract involvement is suspected (Annese et al., 2013). However, UGI tract involvement is a diagnostically challenging presentation in CD, due to a lack of specific clinical symptoms, and thus, there is a heavier reliance on imaging modalities in practice.

Chronic inflammation in CD patients is related to altered interactions between the host and the microbiota, and microbial imbalance (Frank et al., 2007; Xavier and Podolsky, 2007; Sartor, 2008; Halfvarson et al., 2017). Currently, the human microbiome, comprising of the entire microbial complement related with human hosts, is a critical and emerging area for biomarker discovery (Pascal et al., 2017; Douglas et al., 2018; Mills et al., 2019). The identification of microbial biomarkers and their use for the prediction of the disease provide valuable information for predictions in a wide range of applications.

Hence, the aims of this study were to compare the metagenomic profile in CD patients with and without UGI involvement at diagnosis, and to identify the metagenomic biomarkers predicting its development.

## Materials and Methods

### Data Sources and Processing

We used the data from the Inflammatory Bowel Disease Multi’omics Database^1^ for the most comprehensive description to date of host and microbial activities in inflammatory bowel diseases. Tissue samples gathered during the initial screening colonoscopy at diagnosis were collected according to a standardized protocol, and the V4 region of the 16S rRNA gene was PCR-amplified and sequenced in the MiSeq platform (Illumina) (for detailed protocols see http://ibdmdb.org/protocols). We divided the subjects into two groups, “nonL4” versus “L4” -where nonL4 are CD patients without UGI tract involvement and L4 are those with UGI tract involvement in disease extent.

### Community Analysis

The obtained raw data were analyzed using Quantitative Insights Into Microbial Ecology (QIIME) version 1.9.0, a software that performs microbial community analysis and taxonomic classification of microbial genomes (Navas-Molina et al., 2013). Sequences were assigned to operational taxonomic units (OTUs) with a 97% similarity threshold and subsequently picked by UCLUST against a closed reference table, the latest version of the Greengenes OTU database (Edgar, 2010). For diversity analysis, samples were normalized so all the samples could be compared. Alpha diversity of OTU libraries was described using the Chao1, phylogenetic diversity (PD) whole tree, and observed species, and were compared using a Student’s *t*-test. Distance matrices were constructed using the unweighted and weighted UniFrac algorithms in QIIME from the whole community phylogenetic tree. Significant differences between the predefined groups were analyzed using one-way analysis of similarities (ANOSIM) with 999 permutations with their corresponding Global-R statistics.

### Biomarker Detection and Functional Analysis

To determine the potential biomarker OTUs, linear discriminant analysis effect size (LEfSe) analysis was performed with a linear discriminant analysis (LDA) score threshold of > 1.0 to detect features significantly different in abundance between the groups (Fisher, 1936; Segata et al., 2011).

In addition, we conducted subsequence-based 16S rRNA data processing using the DiTaxa software, which substitutes standard OTU-clustering method by segmenting 16S rRNA reads into the most frequent variable-length subsequences, for sequence phenotype classification and biomarker detection (Asgari et al., 2018). The linear support vector machine (SVM) and random forest classifier (RF) algorithms were used to build a predictive model and to calculate the importance of all variables and rank them accordingly. For linear SVM, we set the cost to the value of 1 and use RF classifier in the default settings.

PICRUSt2 was used to predict microbial content from each sample’s data and functionally annotate the data (Langille et al., 2013). The results were further subjected to statistical analysis of taxonomic and functional profiles (STAMP v2.1.1) software (Parks et al., 2014). To investigate the metabolic network of the predicted organism, we used MetaCyc database^2^, which contains data regarding chemical compounds, reactions, enzymes, and metabolic pathways that have been experimentally validated and reported in the scientific literature (Caspi et al., 2016). The statistical analyses were performed using R version 3.5.1 (R Core Team, 2017; Venables and Smith, 2020). All significant thresholds were set at a two-sided *p*-value of 0.05.

## Results

### Baseline Characteristics

Among the 37 potential CD patients, four patients with insufficient data on disease extent and seven patients who were not receiving tissue samples at the time of diagnosis were excluded, leaving 26 patients for analysis. Patients of the L4 group were diagnosed at a younger median age of 13.0 years (IQR 10.5–15.5 years) compared to 19.0 years (IQR 14.5–28.0 years) for the patients in nonL4 group (*P* = 0.005), and the male to female ratio was 2.3 and 1.2 in the L4 and nonL4 groups, respectively (*P* = 0.650) (Table 1). The baseline C-reactive protein (CRP) score and simple endoscopic score for Crohn’s disease (SES-CD) did not differ significantly between the groups (*P* = 0.711 and *P* = 0.056 for L4 and nonL4, respectively) (Table 1). However, the CD patients with UGI tract involvement had higher erythrocyte sedimentation rate (ESR) than those without UGI tract involvement (*P* = 0.033) (Table 1). All tissue samples were obtained from the rectum and ileum (Table 1). None of the patients were on any active medication, such as corticosteroids, immunomodulators, or biological agents at the time of sample collection. The detailed demographic and clinical characteristics are summarized in Table 1.

**TABLE 1:** Baseline characteristics of the patients.

Variable	L4 (*n* = 13)	nonL4 (*n* = 13)	*P*-value
Age at diagnosis, year (median, IQR)	13.0 (5.0)	19.0 (13.5)	0.005
Race (*n*, %)			1.000
White	12 (92.3)	12 (92.3)	
American, Indian, or Alaska Native	1 (7.7)	0 (0.0)	
Other	0 (0.0)	1 (7.7)	
Sex (n, %)			0.650
M	9 (69.2)	7 (53.8)	
F	4 (30.8)	6 (46.2)	
Biopsy location (*n*, %)			0.216
Ileum	6 (46.2)	3 (23.1)	
Rectum	7 (53.8)	10 (76.9)	
ESR (mm/h)	40.0 (27.0)	11.0 (28.0)	0.033
CRP (mg/L)	4.8 (3.3)	4.9 (12.6)	0.711
SES-CD score	8 (9.0)	2.5 (6.0)	0.056

*IQR, interquartile range; ESR, erythrocyte sedimentation rate; CRP, C-reactive protein, SES-CD, simple endoscopic score for Crohn’s disease. The *p*-Values were obtained using Mann–Whitney test and chi-square test.*

### Taxonomic Characterization

We analyzed the intestinal microbiota diversity of the two groups and tested whether intestinal microbiota diversity could be related to disease extent. The alpha diversity indices of Chao1, PD whole tree, and observed species diversity are shown in Supplementary Figure S1. All three diversity indices were higher in nonL4 compared to L4, but there were no significant differences between the two groups (*P* = 0.522, *P* = 0.503, and *P* = 0.275 for Chao1, PD whole tree, and observed species diversity, respectively; Supplementary Figure S1). Beta diversity was further evaluated using weighted-UniFrac analysis, which showed similar bacterial communities in patients of both groups (Supplementary Figure S1). Furthermore, an unweighted UniFrac-based principal coordinate analysis (PCoA) showed that samples were clustered by subject (ANOSIM: *R* = −0.010; *P* = 0.477) (Supplementary Figure S2). We also performed a weighted-UniFrac PCoA analysis with ANOSIM (*R* = −0.043; *P* = 0.898) (Supplementary Figure S2).

### Bacterial Abundance and Distribution

**FIGURE 1:**
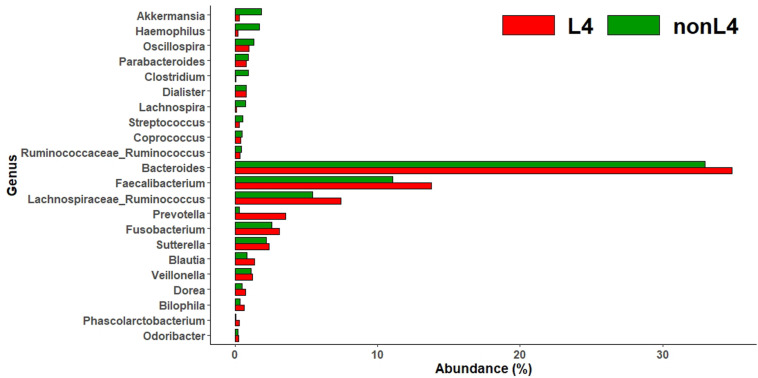
Genus-level taxonomic distribution of intestinal microbiota and top 22 genera in L4 versus nonL4 groups.

Subsequently, we analyzed the intestinal microbiota abundance and distribution in the two groups and tested whether they could be related to UGI tract involvement in CD. At the genus level, bacteria from *Akkermansia* (0.3% vs. 1.8%), *Haemophilus* (0.2% vs. 1.7%), *Oscillospira* (1.0% vs. 1.3%), *Parabacteroides* (0.8% vs. 0.9%), *Clostridium* (0.1% vs. 0.9%), *Dialister* (0.7% vs. 0.8%), *Lachnospira* (0.1% vs. 0.7%), *Streptococcus* (0.3% vs. 0.5%), *Coprococcus* (0.4% vs. 0.5%), and *Ruminococcus* [f__Ruminococcaceae] (0.3% vs. 0.4%) were less abundant, whereas those from *Bacteroides* (34.9% vs. 33.0%), *Faecalibacterium* (13.7% vs. 11.1%), *Ruminococcus* [f__Lachnospiraceae] (7.4% vs. 5.4%), *Prevotella* (3.5% vs. 0.3%), *Fusobacterium* (3.1% vs. 2.6%), *Sutterella* (2.4% vs. 2.2%), *Blautia* (1.3% vs. 0.8%), *Veillonella* (1.2% vs. 1.1%), *Dorea* (0.7% vs. 0.5%), *Bilophila* (0.6% vs. 0.3%), *Phascolarctobacterium* (0.3% vs. 0.1%), and *Odoribacter* (0.2% vs. 0.1%) were more abundant in L4 compared to nonL4 (Figure 1).

### Metagenomic Biomarker Discovery

**FIGURE 2:**
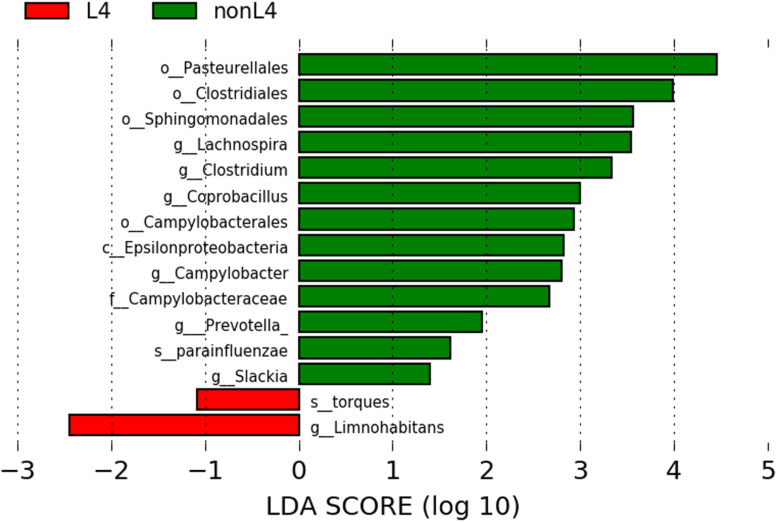
Histogram of the LDA scores computed for features with differential abundance in L4 (red) and nonL4 (green) groups. Horizontal bars represent the effect size for each taxon. The length of the bar represents the log10 transformed LDA score, indicated by vertical dotted lines. The threshold on the logarithmic LDA score for discriminative features was set to 1.0. The taxon of bacteria with statistically significant change (*P* < 0.05) in the relative abundance is written alongside the horizontal lines. The name of the taxon level is abbreviated as c—class; o—order; f—family; g—genus; and s—species.

We found significant differences in the community compositions between the two groups by LEfSe analysis. As shown in Figure 2, the microbial composition was also significantly different at the order level among groups. The Pasteurellales (*P* = 0.042), Sphingomonadales (*P* = 0.045), Campylobacterales (*P* = 0.024), and Clostridiales (*P* = 0.043) exhibited a relatively higher abundance in nonL4 group (Figure 2). The patients in nonL4 group had members of the class Epsilonproteobacteria (*P* = 0.024) and the family *Campylobacteraceae* (*P* = 0.024) that were significantly dominant than those in L4 group patients (Figure 2). Furthermore, there were seven significantly different genera, composed of *Campylobacter* (*P* = 0.024), *Prevotella* (*P* = 0.034), *Clostridium* (*P* = 0.043), *Coprobacillus* (*P* = 0.015), *Slackia* (*P* = 0.034), and *Lachnospira* (*P* = 0.015) that were enriched in the nonL4 group, while *Limnohabitans* (*P* = 0.034) was enriched in the L4 group (Figure 2). At the species level, significantly more *Haemophilus parainfluenzae* were detected in the patients in nonL4 group (*P* = 0.028), while *Ruminococcus torques* were enriched in L4 group patients (*P* = 0.015) (Figure 2).

Comparative taxonomic visualization of detected differentially expressed markers for DiTaxa and a common workflow are shown in Supplementary Figure S3 for samples from CD patients with UGI tract involvement versus those without UGI tract involvement. Taxa predicted by DiTaxa analysis for samples from L4 group patients versus those from nonL4 group exhibited *Ruminococcus faecis*, *Coprococcus comes*, *Dorea formicigenerans*, *Ruminococcus torques*, CCMM_s, *Eubacterium hallii*, *Bilophila wadsworthia*, *Blautia faecis*, *Ruminococcus gnavus*, *Alistipes putredinis*, *Bacteroides finegoldii*, *Roseburia faecis*, *Faecalibacterium prausnitzii*, *Roseburia inulinivorans*, *Lachnospira pectinoschiza*, *Intestinibacter bartlettii*, *Clostridium symbiosum*, *Agathobacter rectalis*, *Roseburia intestinalis*, *Clostridium bolteae, Fusicatenibacter saccharivorans, Anaerostipes hadrus, Bacteroides caccae, Bacteroides uniformis*, *Flavonifractor plautii* as significantly associated (Table 2).

**TABLE 2:** Taxa predicted by DiTaxa analysis for UGI tract involvement in CD.

**Direction**	**Taxonomy**	**Marker**	***P*-value**	**Number of markers**
+	*Ruminococcus faecis*	tacgtatggtgcaagcgttatccggatttactgggtgtaaagggagcgtagacggagtggcaagtctggtgtgaaaacccggggct caaccccgggactgcattggaaactgtcaatctagagtaccggagaggtaagcggaattcctagtgtagcggtgaaatgcgtagat attaggaggaacaccagtggcgaaggcggcttactggacggtaactgacgttgaggctcgaaagcgtggggagcaaacagg	0.014	2
+	*Coprococcus comes*	tacgtatggtgcaagcgttatccggatttactgggtgtaaagggagcgtagacggctgtgtaagtctgaagtgaaagcccggggctc aaccccgggactgctttggaaactatgcagctagagtgtcggagaggtaagtggaattcccagtgtagcggtgaaatgcgtagatatt gggaggaacaccagtggcgaaggcggcttactggacggtaactgacgttgaggctcgaaagcgtggggagcaaacagg	0.025	1
+	Dorea formicigenerans*	tacgtatggtgcaagcgttatccggatttactgggtgtaaagggagcgtagacggctgtgcaagtctgaagtgaaaggcatgggctca acctgtggactgctttggaaactgtgcagctagagtgtcggagaggtaagtggaattcctagtgtagcggtgaaatgcgtagatattag gaggaacaccagtggcgaaggcggcttactggacgatgactgacgttgaggctcgaaagcgtggggagcaaacagg	0.025	1
+	Ruminococcus torques*	tacgtatggtgcaagcgttatccggatttactgggtgtaaagggagcgtagacggagtggcaagtctgatgtgaaaacccggggctc aaccccgggactgcattggaaactgttcatctagagtgctggagaggtaagtggaattcctagtgtagcggtgaaatgcgtagatatta ggaggaacaccagtggcgaaggcggcttactggacagtaactgacgttgaggctcgaaagcgtggggagcaaacagg	0.025	1
+	CCMM s	tacgtaggtggcgagcgttatccggaattattgggcgtaaagagggagcaggcggcactaagggtctgtggtgaaagatcgaagc ttaacttcggtaagccatggaaac	0.025	1
+	*Eubacterium hallii*	tgctcggctagagtacaggagaggcaggcggaattcctagtgtagcggtgaaatgcgtagatattaggaggaacaccagtggcg aagcgggcctgctggactgttactgacgctgaggcacgaaagcgtggggagcaaacagg	0.046	1
+	*Bilophila wadsworthia*	tccgtagatatctggaggaacaccggtggcgaaggcggccacctggacggtaactgacgctgaggtgcgaaagcgtgggtagc aaacagg	0.046	1
+	*Blautia faecis*	tacgtagggggcaagcgttatccggatttactgggtgtaaagggagcgtagacggcgcagcaagtctgatgtgaaaggcagggg cttaacccctggactgcattggaaactgctgtac	0.046	1
+	Ruminococcus gnavus*	tacgtagggggcaagcgttatccggatttactgggtgtaaagggagcgtagacggcatggcaagccagatgtgaaagcccggggc tcaaccccgggactgcatttggaactgtcaggctagagtgtcggagaggaaagcggaattcctggtgtagcggtgaaatgcgtagat attaggaggaacaccagtggcgaaggcggctttctggacgatgactgacgttgaggctcgaaagcgtggggagcaaacagg	0.046	1
+	*Alistipes putredinis*	tacggaggattcaagcgttatccggatttattgggtttaaagggtgcgtaggcggtttgataagttagaggtgaaatttcggggctcaa ccctgaacgtgcctctaatactgttgagctagagagtagttgcggtaggcggaatgtatggtgtagcggtgaaatgcttagagatcat acagaacaccgattgcgaaggcagcttaccaaactatacctgacgttgaggcacgaaagcgtggggagcaaacagg	0.046	1
+	*Bacteroides finegoldii*	tacggaggatccgagcgttatccggatttattgggtttaaagggagcgtaggtggattgttaagtcagttgtgaaagtttgcggctca accgtaaaattgcagttgatactggctgtcttgagtacagtagaggtgggcgg	0.046	1
−	Roseburia faecis*	tacgtatggtgcaagcgttatccggatttactgggtgtaaagggagcgcaggcggtgcggcaagtctgatgtgaaagcccggggctca accccggtactgcattggaaactgtcgtactagagtgtcggaggggtaagtggaattcctagtgtagcggtgaaatgcgtagatatta ggaggaacaccagtggcgaaggcggcttactggacgataact gacgctgaggctcgaaagcgtggggagcaaacagg	0.005	1
−	Faecalibacterium prausnitzii*	aaggcaagttggaagtgaaatccatgggctcaacccatgaactgctttcaaaactgtttttcttgagtagtgcagaggtaggcggaat tcccggtgtagcggtggaatgcgtagatatcgggaggaacaccagtggcgaaggcggcctactgggcaccaactgacgctga ggctcgaaagtgtgggtagcaaacagg	0.014	1
−	*Roseburia inulinivorans*	tacgtatggtgcaagcgttatccggatttactgggtgtaaagggagcgcaggcggaaggctaagtctgatgtgaaagcccggggct caaccccggtac	0.025	1
−	*Lachnospira pectinoschiza*	agaggcaagtggaattcctagtgtagcggtgaaatgcgtagatattaggaggaacaccagtggcgaaggcggcttgctggactgtaa ctgacactgaggctcgaaagcgtggggagcaaacagg	0.025	1
−	*Intestinibacter bartlettii*	tacgtagggggctagcgttatccggatttactgggcgtaaagggtgcgtaggcggtcttttaagtcaggagtgaaaggctacggctca accgtagtaagctcttgaaactggaggacttgagtgcaggagaggagagtggaattcctagtgtagcggtgaaatgcgtagatattag gaggaacaccagtagcgaaggcggctctctggactgtaactgacgctgaggcacgaaagcgtggggagcaaacagg	0.035	1
−	*Roseburia inulinivorans*	tacgtatggtgcaagcgttatccggatttactgggtgtaaagggagcgcaggcggagggctaagtctgatgtgaaagcccggggc tcaaccccggtactgcattggaaactggtcatctagagtgtcggaggggtaagtggaattcctagtgtagcggtgaaatgcgtaga tattaggaggaacaccagtggcgaaggcggcttactggacgataactgacgctgaggctcgaaagcgtggggagcaaacagg	0.046	5
−	*Clostridium symbiosum*	tgtttaactggagtgtcggagaggtaagtggaattcctagtgtagcggtgaaatgcgtagatattaggaggaacaccagtggcgaa ggcgacttactggacgataactgacgttgaggctcgaaagcgtggggagcaaacagg	0.046	1
−	*Agathobacter rectalis*	tacgtatggtgcaagcgttatccggatttactgggtgtaaagggagcgcaggcggtgcggcaagtctgatgtgaaagcccggggct caaccccggtactgcattggaaactgtcgtactagagtgtcggaggggtaagcggaattcctagtgtagcggtgaaatgcgtagatat taggaggaacaccagtggcgaaggcggcttactggacgataactgacactgaggctcgaaagcgtggggagcaaacagg	0.046	1
−	*Roseburia intestinalis*	tacgtatggtgcaagcgttatccggatttactgggtgtaaagggagcgcaggcggtacggcaagtctgatgtgaaagcccggggct caaccccggtactgcattggaaactgtcggac	0.046	1
−	*Clostridium bolteae*	tacgtaggtggcaagcgttatccggatttactgggtgtaaagggagcgtagacggcgaagcaagtctgaagtgaaaacccagggc tcaaccctgggactgctttggaaactgttttgctagagtgtcggagaggtaagtggaattcctagtgtagcggtgaaatgcgtagat attaggaggaacaccagtggcgaaggcggcttactggacgataactgacgttgaggctcgaaagcgtggggagcaaacagg	0.046	1
−	*Fusicatenibacter saccharivorans*	tacgtagggggcaagcgttatccggatttactgggtgtaaagggagcgtagacggcaaggcaagtctgatgtgaaaacccaggg cttaaccctgggactgcattggaaactgtctggctcgagtgccggagaggtaagcggaattcctagtgtagcggtgaaatgcgtaga tattaggaagaacaccagtggcga	0.046	1
−	*Anaerostipes hadrus*	tacgtagggggcaagcgttatccggaattactgggtgtaaagggtgcgtaggtggtatggcaagtcagaagtgaaaacccaggg cttaactctgggactgcttttgaaactgtcagactggagtgcaggagaggtaagcggaattcctagtgtagcggtgaaatgcgtagat attaggagg	0.046	1
−	Bacteroides caccae*	tacggcggatccgagcgttatccggatttattgggtttaaagggagcgtaggcggattgttaagtcagttgtgaaagtttgcggctcaac cgtaaaattgcagttgatactggcagtcttgagtgcagtagaggtgggcggaattcgtggtgtagcggtgaaatgcttagatatcacg aagaactccgattgcgaaggcagctcactggagtgtaactgacgctgatgctcgaaagtgtgggtatcaaacagg	0.046	1
−	*Bacteroides caccae*	tacggaggatccgagcgttatccggatttattgggtttaaagggagcgtaggcggattgttaagtcagttgtgaaagtttgcggctcaa ccgtaaaattgcagttgatactggcagtcttgagtgcagtagaggtgggcggaattcgtggtgtagcggtgaaatgcttagatatcacga agaactccgattgcggaggcagctcactggagtgtaactgacgctgatgctcgaaagtgtgggtatcaaacagg	0.046	1
−	Bacteroides uniformis*	tacggaggatccgagcgttatccggatttattgggtttaaagggagcgtaggcggacgcttaagtcagttgtgaaagtttgcggctcaa ccgtaaaattgcagttgatactgggtgtcttgagtacagtagaggcaggcggaattcgtggtgtagcggtgaaatgcttagatat cacgaagaactccgattgcgaaggcagcctgctggactgtaactgacgctgatgctcgaaagtgtgggtatcaaaaagg	0.046	1
−	*Flavonifractor plautii*	taaagggcgtgtaggcgggattgcaagtcagatgtgaaaactgggggctcaacctccagcctgcatttgaaactgtagttc	0.046	1

UGI, upper gastrointestinal; CD, Crohn’s disease; *+*, prediction for L4 group; −, prediction for nonL4 group; *taxa identified with UCLUST-based methods in QIIME.

Of these, the following seven taxa were actually identified with UCLUST-based methods in QIIME (Table 2): *Dorea formicigenerans*, *Ruminococcus torques*, *Ruminococcus gnavus*, *Roseburia faecis*, *Faecalibacterium prausnitzii*, *Bacteroides caccae*, and *Bacteroides uniformis*.

### Metagenomic Biomarker Evaluation

**FIGURE 3:**
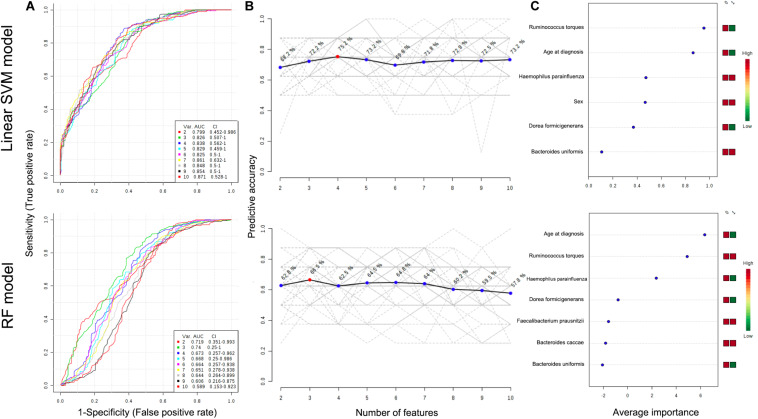
ROC curves for combination model calculated from the linear SVM and RF models **(A)**. The number of features optimizing the performance of the models **(B)**. Features ranked by their contributions to classification accuracy **(C)**. The features are ranked by their frequencies of being selected as the classifiers. The colored boxes on the right indicate the relative abundance ratio of the corresponding factor in each group (Group label: nonL4 = 0, L4 = 1).

To further characterize the predictive value of the eight identified taxa by LEfSe or DiTaxa methods, we performed ROC analysis with clinical variables (age at diagnosis and sex) using the machine learning models (Figure 3). A comparison of the average performance as a predictive model suggests the superiority of SVM: the average performance of SVM is >0.799 AUC and 68.2–75.2% accuracy, while that of RF is <0.740 AUC and 57.8–66.5% accuracy (Figure 3). For the top performing model architecture, the addition of microbial features improves the predictive performance of linear SVM model; however, the performance in RF model tends to decrease. Notably, for the feature ranking method in both linear SVM and RF models, the top two factors -the species *Ruminococcus torques* and age at diagnosis- contributed to the combined models (Figure 3). Figure 3 also shows that the addition of the signature of the species *Haemophilus parainfluenzae* into the models enabled us to achieve the highest accuracy and to increase the diagnostic performance of UGI tract involvement in CD patients.

### Metagenomic Functional Analysis

**FIGURE 4:**
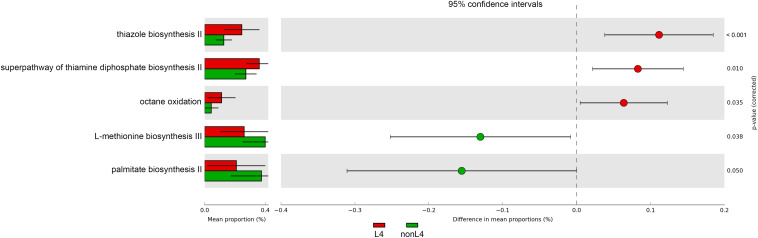
Prediction of changed KEGG pathways using PICRUSt2 analysis. A total of five KEGG pathways were significantly altered in the L4 group compared to the nonL4 group; Bar plots on the left side display the mean proportion of each KEGG pathway. Dot plots on the right show the differences in mean proportions between the two indicated groups using *P*-values.

**FIGURE 5:**
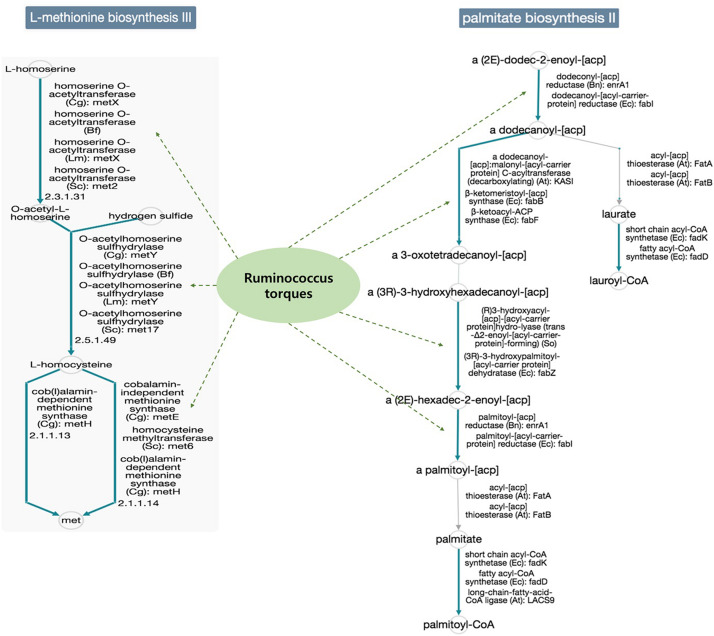
Functional annotation of predicted species in the elucidated pathways represented in MetaCyc (solid blue line: general reactions, solid gray line: spontaneous or missing reactions, dotted green line: reaction step predicted for the species). Potential mechanisms implicated in the interplay between the gut microbiomes would be influenced the UGI involvement in CD, because, each reaction step in the pathways was not enriched only for the species *Ruminococcus torques* in the database.

In addition, the functional diversity of the different putative metagenomes was assessed using the PICRUSt2 software. Pathways displaying a significant difference in mean proportions between L4 and nonL4 groups were represented (Figure 4). The pathways, including thiazole biosynthesis II (*p* < 0.001), superpathway of thiamine diphosphate biosynthesis II (*P* = 0.010), and octane oxidation (*P* = 0.035), were over-represented, whereas L-methionine biosynthesis III (*P* = 0.038) and palmitate biosynthesis II (*P* = 0.050) were under-represented in L4 (Figure 4). For selected pathways, we also examined the extent to which these pathways are linked with the species *Ruminococcus torques*. As shown in Figure 5, the two related MetaCyc pathways, L-methionine biosynthesis III and palmitate biosynthesis II, showed an association with *Ruminococcus torques*, which may play important roles in the intestinal integrity and barrier function (Figure 5).

## Discussion

To our knowledge, this is the first study to identify a reliable metagenomic biomarker for UGI tract involvement in CD. The reported frequency of UGI tract involvement in CD largely varies. The main cause of the discrepancies regarding prevalence rates of UGI tract lesions is probably related to irregularly performing different diagnostic modalities for CD diagnosis, presumably because of the low reliability of mapping disease extent in clinical practice. However, to date, no data has been analyzed regarding a simple biomarker for CD patients with UGI tract involvement.

Our main hypothesis is that the possible differences in taxonomic composition might potentially be used as proxy biomarkers for UGI tract involvement in CD patients, since altered microbial communities have been demonstrated to be an essential factor in driving intestinal inflammation in CD (Tamboli et al., 2004; Sartor, 2008).

We analyzed the differences in the tissue microbial community of CD patients at the species level. In this study, the species *Dorea formicigenerans*, *Ruminococcus torques*, *Ruminococcus gnavus*, *Roseburia faecis*, *Faecalibacterium prausnitzii*, *Bacteroides caccae*, *Bacteroides uniformis*, and *Haemophilus parainfluenza* were identified as predictive biomarkers by the LEfSe or DiTaxa programs. Interestingly, *Ruminococcus torques*, a butyrate-producing bacterial species, was the only one commonly identified by the two different algorithms.

The authors also examined whether the composition of the microbiota, with clinical predictors, could predict whether the patient would have UGI tract involvement or not using two different machine learning algorithms (linear SVM and RF). Modest predictive performances were achieved with a few features (eight taxa, age at diagnosis, and sex), especially in linear SVM. Notably, the most influential features for predicting disease extent were levels of the species *Ruminococcus torques* (positive correlation) and age at diagnosis (negative correlation). These consistent reproducible results for the species *Ruminococcus torques* present the possibility of using microbiota analysis as a screening tool to determine CD patients at high risk of UGI tract involvement.

Contrary to the inconsistent results regarding the signature of *Ruminococcus torques* in fecal samples of CD patients (Joossens et al., 2011; Gevers et al., 2014; Takahashi et al., 2016), most of the studies from tissue samples showed consistently high levels of the species in the mucosa of patients with CD compared to that in healthy subjects (Martinez-Medina et al., 2006; Png et al., 2010). Furthermore, our results show that its abundance remains significantly high in the CD patients with UGI tract involvement.

No role in CD pathophysiology has been suggested so far for the species *Ruminococcus torques*, belonging to the *Clostridium coccoides* group/cluster XIVa. They utilize MUC2, the main secreted mucin in the human intestine, as the sole carbon source and have a strong gastrointestinal mucin-degrading ability, providing further evidence of their adaptability in the human gut mucosal environment (Colina et al., 1996; Dethlefsen et al., 2006; Png et al., 2010). Therefore, it has been proposed that excessive mucin degradation by these bacteria may contribute to intestinal disorders, as access of luminal antigens to the intestinal immune system is facilitated (Ganesh et al., 2013).

In addition, we found an inverse relationship for age at diagnosis with UGI involvement in CD. Present study showed that the median age of the patients with UGI involvement was significantly lower compared to those without UGI involvement. This is in accordance with a previous study by Thomas and colleagues who demonstrated a higher rate of younger patients (≤16 years) suffering from UGI tract involvement compared to those without (9.4% versus 17.8%, *P* = 0.005) (Greuter et al., 2018). Another study by Lopez-Siles et al. observed that CD patients below 16 years of age had a striking reduction in the population of *Akkermansia* sp. that possess a lower mucolytic activity compared to those with disease onset at a later age, which is in agreement with our findings (Lopez-Siles et al., 2018).

Taken together, the mucus barrier dysfunction, due to the replacement of a less mucolytic bacteria, such as *Akkermansia* species by a more mucolytic one, such as *Ruminococcus torques*, at young age may influence the microbial community on the intestinal mucosa and be instrumental in the development of UGI tract involvement in CD.

To characterize the functional role of the microbiome in phenotype, we annotated the taxa by the Kyoto Encyclopedia of Genes and Genomes (KEGG) database. This analysis suggested that L-methionine biosynthesis III and palmitate biosynthesis II pathway were decreased, which are linked with *Ruminococcus torques*, while the three KEGG pathways predicted to be increased in L4 group were not associated with the species. Currently, the role of methionine metabolism and its metabolites, and palmitate metabolic pathway in the pathogenesis of CD is poorly understood. Methionine is known to improve the integrity and barrier function of the small intestinal mucosa and villus morphology, and development in previous studies from animal models (Chen et al., 2014; Shen et al., 2014). A previous *in vivo* study by Wei et al. showed that palmitate plays a key role in the preservation of the gut barrier function by regulating the secretion and function of MUC2 (Wei et al., 2012). Another recent study also demonstrated that palmitate enhances MUC2 production in goblet cells of intestine, leading to the establishment of a thick mucus gel, thereby maintaining the integrity of the gut barrier (Benoit et al., 2015). These findings imply that there might be a decrease in the two protective pathways through the communication between intestinal cells and microbial community, especially the species *Ruminococcus torques* may induce excessive mucus degradation of small intestine in CD patients with UGI tract involvement.

The main strength of this study is that it evaluated the potential metagenomic biomarkers for prediction of UGI tract involvement in CD patients through various analyses. Further, we analyzed the CD patients with new-onset disease, before the commencement of treatment. Changes in microbiota community structure important for disease pathogenesis are likely to be more evident in new-onset and treatment-naive patients than those undergoing treatment. Lastly, the study focuses on the mucosa-associated microbiota samples, which may be more relevant to disease pathogenesis and diagnosis than fecal samples. However, our study was limited by the small sample size. Another limitation was that it comprised of predominately white patients, and thus, the findings may not be generalized to other racial populations. Finally, although our study was focused only on the microbial community, microbial metabolites also have great potential for improving diagnosis of CD and reflect the abnormalities of the host intestine microbiota. Therefore, new biomarkers for CD patients with UGI involvement could be developed by integrated analysis of metabolomics and metagenomics from a multinational and multicenter cohort.

## Conclusion

In conclusion, the species *Ruminococcus torques* in the tissue microbial community of CD patients might serve as a novel potential biomarker for UGI tract involvement. The UGI tract involvement in CD is higher in younger age group patients; therefore, it should be carefully monitored in them. The mechanisms of interactions between the host and *Ruminococcus torques* should be further investigated.

## Data Availability Statement

All datasets presented in this study are included in the article/Supplementary Material.

## Ethics Statement

Ethical review and approval was not required for the study on human participants in accordance with the local legislation and institutional requirements. Written informed consent from the participants’ legal guardian/next of kin was not required to participate in this study in accordance with the national legislation and the institutional requirements.

## Author Contributions

MK designed the study. JC and HS analyzed and interpreted the data, and wrote the manuscript. JJ and JY supervised the project and revised the manuscript. All authors vouch for the data and analysis, have approved the final version, and agreed to publish the manuscript.

## Conflict of Interest

The authors declare that the research was conducted in the absence of any commercial or financial relationships that could be construed as a potential conflict of interest.

## Footnotes

1 http://ibdmdb.org

2 https://MetaCyc.org

## Supplementary Material

FIGURE S1 Analysis of alpha diversity as predicted by Chao 1 estimator, PD whole tree, and observed species **(A)**; and beta diversity measured by weighted-UniFrac distances in L4 versus nonL4 groups **(B)**.

FIGURE S2 Principal coordinates analysis (PCoA) based on **(A)** unweighted and **(B)** weighted UniFrac distance; blue for the nonL4 and red for the L4 (ANOSIM: *R* = −0.010, *P* = 0.477; *R* = −0.043, *P* = 0.898).

FIGURE S3 Heat map of occurrence of markers by DiTaxa analysis between L4 and nonL4 groups. The rows are sorted based on the taxonomic marker assignments and the columns represent each group and are sorted firstly, based on their phenotype, and secondly, based on their pattern similarity.
