# Supplementary figures and images for: Development of a Novel Metagenomic Biomarker for Prediction of Upper Gastrointestinal Tract Involvement in Patients With Crohn’s Disease
Source: Front Microbiol. 2020 Jun 3;11:1162. doi: 10.3389/fmicb.2020.01162 (PMC7283919; doi:10.3389/fmicb.2020.01162)

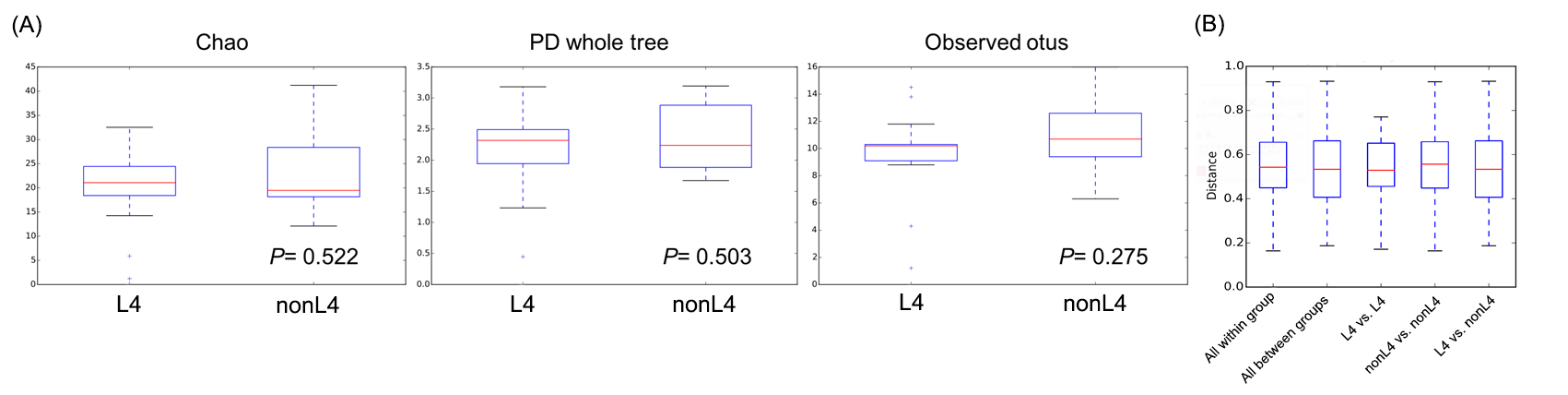

Supplement: FIGURE S1 — Analysis of alpha diversity as predicted by Chao 1 estimator, PD whole tree, and observed species (A); and beta diversity measured by weighted-UniFrac distances in L4 versus nonL4 groups (B). [file Image_1.TIF]

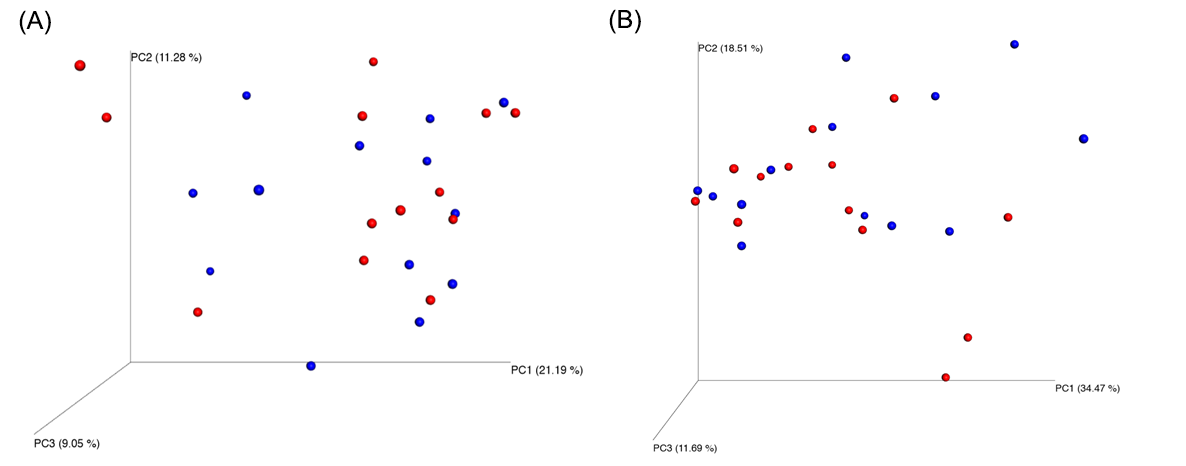

Supplement: FIGURE S2 — Principal coordinates analysis (PCoA) based on (A) unweighted and (B) weighted UniFrac distance; blue for the nonL4 and red for the L4 (ANOSIM: R = −0.010, P = 0.477; R = −0.043, P = 0.898). [file Image_2.TIF]
