# Supplementary material for: Development of a Novel Metagenomic Biomarker for Prediction of Upper Gastrointestinal Tract Involvement in Patients With Crohn’s Disease
Source: Front Microbiol. 2020 Jun 3;11:1162. doi: 10.3389/fmicb.2020.01162 (PMC7283919; doi:10.3389/fmicb.2020.01162)
Supplement: FIGURE S3 — Heat map of occurrence of markers by DiTaxa analysis between L4 and nonL4 groups. The rows are sorted based on the taxonomic marker assignments and the columns represent each group and are sorted firstly, based on their phenotype, and secondly, based on their pattern similarity. [file Data_Sheet_1.pdf]

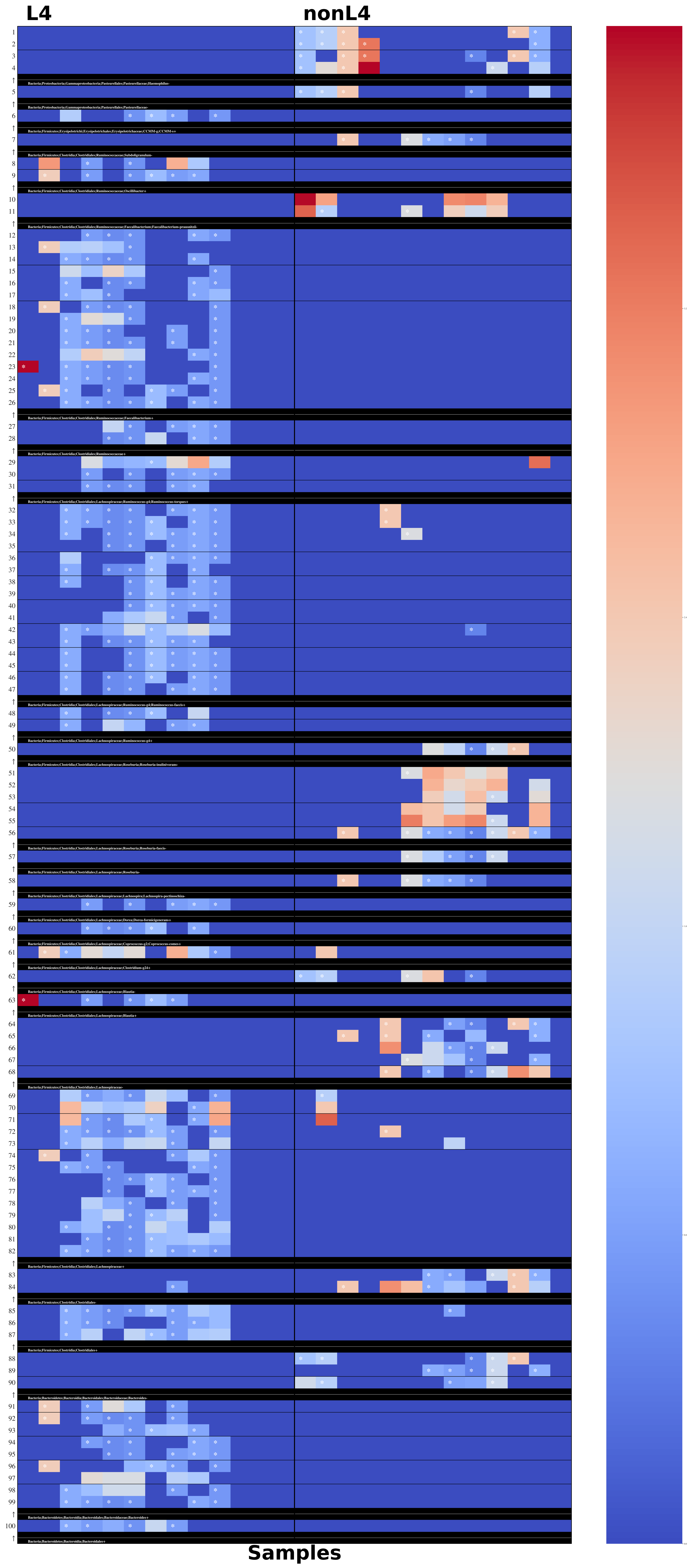

L4
nonL4
Bacteria;Proteobacteria;Gammaproteobacteria;Pasteurellales;Pasteurellaceae;Haemophilus-
Bacteria;Proteobacteria;Gammaproteobacteria;Pasteurellales;Pasteurellaceae-
Bacteria;Firmicutes;Erysipelotrichi;Erysipelotrichales;Erysipelotrichaceae;CCMM-g;CCMM-s+
Bacteria;Firmicutes;Clostridia;Clostridiales;Ruminococcaceae;Subdoligranulum-
Bacteria;Firmicutes;Clostridia;Clostridiales;Ruminococcaceae;Oscillibacter+
Bacteria;Firmicutes;Clostridia;Clostridiales;Ruminococcaceae;Faecalibacterium;Faecalibacterium-prausnitzii-
Bacteria;Firmicutes;Clostridia;Clostridiales;Ruminococcaceae;Faecalibacterium+
Bacteria;Firmicutes;Clostridia;Clostridiales;Ruminococcaceae+
Bacteria;Firmicutes;Clostridia;Clostridiales;Lachnospiraceae;Ruminococcus-g4;Ruminococcus-torques+
Bacteria;Firmicutes;Clostridia;Clostridiales;Lachnospiraceae;Ruminococcus-g4;Ruminococcus-faecis+
Bacteria;Firmicutes;Clostridia;Clostridiales;Lachnospiraceae;Ruminococcus-g4+
Bacteria;Firmicutes;Clostridia;Clostridiales;Lachnospiraceae;Roseburia;Roseburia-inulinivorans-
Bacteria;Firmicutes;Clostridia;Clostridiales;Lachnospiraceae;Roseburia;Roseburia-faecis-
Bacteria;Firmicutes;Clostridia;Clostridiales;Lachnospiraceae;Roseburia-
Bacteria;Firmicutes;Clostridia;Clostridiales;Lachnospiraceae;Lachnospira;Lachnospira-pectinoschiza-
Bacteria;Firmicutes;Clostridia;Clostridiales;Lachnospiraceae;Dorea;Dorea-formicigenerans+
Bacteria;Firmicutes;Clostridia;Clostridiales;Lachnospiraceae;Coprococcus-g2;Coprococcus-comes+
Bacteria;Firmicutes;Clostridia;Clostridiales;Lachnospiraceae;Clostridium-g24+
Bacteria;Firmicutes;Clostridia;Clostridiales;Lachnospiraceae;Blautia-
Bacteria;Firmicutes;Clostridia;Clostridiales;Lachnospiraceae;Blautia+
Bacteria;Firmicutes;Clostridia;Clostridiales;Lachnospiraceae-
Bacteria;Firmicutes;Clostridia;Clostridiales;Lachnospiraceae+
Bacteria;Firmicutes;Clostridia;Clostridiales-
Bacteria;Firmicutes;Clostridia;Clostridiales+
Bacteria;Bacteroidetes;Bacteroidia;Bacteroidales;Bacteroidaceae;Bacteroides-
Bacteria;Bacteroidetes;Bacteroidia;Bacteroidales;Bacteroidaceae;Bacteroides+
Bacteria;Bacteroidetes;Bacteroidia;Bacteroidales+
Samples
